# SNARE Protein Mimicry by an Intracellular Bacterium

**DOI:** 10.1371/journal.ppat.1000022

**Published:** 2008-03-14

**Authors:** Cédric Delevoye, Michael Nilges, Pierre Dehoux, Fabienne Paumet, Stéphanie Perrinet, Alice Dautry-Varsat, Agathe Subtil

**Affiliations:** 1 Institut Pasteur, Unité de Biologie des Interactions Cellulaires, CNRS URA 2582, Paris, France; 2 Institut Pasteur, Unité de Bioinformatique Structurale, CNRS URA 2185, Paris, France; 3 Institut Pasteur, Plate-forme Intégration et Analyse génomique, Paris, France; 4 Department of Physiology and Biophysics, Columbia University Medical Center, New York, New York, United States of America; Duke University Medical Center, United States of America

## Abstract

Many intracellular pathogens rely on host cell membrane compartments for their survival. The strategies they have developed to subvert intracellular trafficking are often unknown, and SNARE proteins, which are essential for membrane fusion, are possible targets. The obligate intracellular bacteria *Chlamydia* replicate within an intracellular vacuole, termed an inclusion. A large family of bacterial proteins is inserted in the inclusion membrane, and the role of these inclusion proteins is mostly unknown. Here we identify SNARE-like motifs in the inclusion protein IncA, which are conserved among most *Chlamydia* species. We show that IncA can bind directly to several host SNARE proteins. A subset of SNAREs is specifically recruited to the immediate vicinity of the inclusion membrane, and their accumulation is reduced around inclusions that lack IncA, demonstrating that IncA plays a predominant role in SNARE recruitment. However, interaction with the SNARE machinery is probably not restricted to IncA as at least another inclusion protein shows similarities with SNARE motifs and can interact with SNAREs. We modelled IncA's association with host SNAREs. The analysis of intermolecular contacts showed that the IncA SNARE-like motif can make specific interactions with host SNARE motifs similar to those found in a bona fide SNARE complex. Moreover, point mutations in the central layer of IncA SNARE-like motifs resulted in the loss of binding to host SNAREs. Altogether, our data demonstrate for the first time mimicry of the SNARE motif by a bacterium.

## Author Summary

Chlamydiae are obligate intracellular bacteria that have co-evolved with eukaryotic cells and adapted to a wide range of hosts, causing several diseases in humans and animals. For example, one species pathogenic to humans, *Chlamydia trachomatis*, is the leading cause of preventable blindness and of bacterial sexually transmitted diseases worldwide. Chlamydiae multiply inside a membrane-bound compartment, the inclusion. The exchanges between the membrane of the inclusion and other intracellular membranes are tightly controlled by the bacteria, for example avoiding fusion with some degradation compartments, while acquiring lipids. Inclusion proteins, made by the bacteria and secreted into the inclusion membrane, are thought to play a central role in controlling these interactions, although their exact function is mostly unknown. We have identified, in three inclusion proteins, a motif common to proteins that are essential for the fusion of two compartments in eukaryotic cells, the SNARE proteins. Via this motif, inclusion proteins interact specifically with a subset of SNAREs of the host, which leads to the selective recruitment of intracellular compartments around the inclusion. This study thus provides a striking example of mimicry of the host by an intracellular pathogen.

## Introduction

*Chlamydia* are obligate intracellular bacterial pathogens of eukaryotic cells. They infect a variety of animals, including humans, and cause acute and chronic diseases [1]–[3]. *Chlamydia* replicate primarily within epithelial cells, in a membrane-bound compartment called the inclusion. The membrane of the inclusion is of dual origin, reflecting its position at the interface between host and pathogen. The bacteria use a type III secretion process to translocate into it the Inc proteins, a large family of *Chlamydia* specific proteins of mostly unknown function [4]–[6]. Host cell proteins might be less abundant in the inclusion membrane, which lacks conventional markers of early and recycling endosomes and avoids fusion with acidic degradative compartments [6],[7]. However, several lines of evidence indicate a contribution of host cell compartments to the inclusion growth [8]–[10]. They suggest that *Chlamydiae* control their interactions with the host intracellular traffic, allowing some fusion events while avoiding others. In eukaryotic cells, SNARE (soluble NSF (N-ethylmaleimide-sensitive factor) attachment protein receptors) proteins play an essential role in compartment fusion [11]. They share a conserved motif, the SNARE motif, and have been classified as Q-SNAREs (glutamine containing SNAREs) and R-SNAREs (arginine containing SNAREs) based on a highly conserved residue at the centre of this motif [12]. SNARE proteins anchored in two lipid bilayers associate in complexes involving three Q-SNARE and one R-SNARE motifs. Complex formation is needed for the fusion of the two lipid bilayers. More recently, it appeared that SNAREs can also have inhibitory role in membrane fusion, by substituting for or binding to a subunit of a fusogenic SNARE bundle to form a nonfusogenic complex [13]. As central regulators of membrane fusion, SNARE proteins appear as possible targets for intracellular organisms, which often rely on subverting the host intracellular traffic. However, although there have been suggestions for the presence of SNARE-like motifs in *Legionnella* effector proteins [14], there is no definite example of mimicry of the SNARE motif by an intracellular bacterium. We have previously shown that one *Chlamydia* inclusion protein might interact with itself to form a complex similar to that of the SNARE complex, and thus facilitate the homotypic fusion of inclusions [15]. This finding led us to hypothesize that one of the functions of some inclusion proteins is to control intracellular trafficking by mimicking SNAREs.

## Results/Discussion

### Identification of SNARE-like motifs in IncA and in other *C. trachomatis* inclusion proteins by bioinformatics

**Figure 1:**
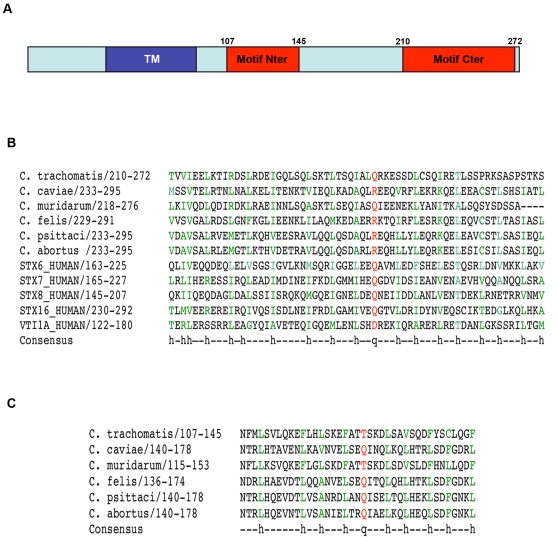
SNARE-like motifs in the inclusion protein IncA. A, Localisation of *C. trachomatis* IncA SNARE-like motifs; TM designates IncA bilobed hydrophobic domain. B, Alignment of IncA SNARE-like motif Cter from 6 *Chlamydia* species and four host SNAREs. The first line indicates *a* and *d* positions in the heptads, conserved hydrophobic residues are shown in green, the conserved Gln or Arg in red. C, Alignment of IncA SNARE-like motif Nter from 6 *Chlamydia* species. Swissprot ID of IncA-like proteins (in the order of the aligned sequences): INCA1_CHLTR, Q46210_CHLCV, Q9PKR8_CHLMU, Q254Q8_CHLFF, Q45SH9_CHLPS, Q5L5U6_CHLAB.

To identify SNARE-like motifs in *Chlamydia* proteins, we used a bioinformatic approach. SNARE motifs are made of heptad repeat sequences that form coil-coiled structures. Position *a* and *d* of the heptads are occupied by hydrophobic residues, except at the centre of the motif, where a very conserved glutamine or arginine residue occupies position *d*, defining the zero layer. Many coiled-coil regions meet these criteria, hampering the identification of SNARE-like motifs from genomic data. However, in this case, we reasoned that the access to several different *Chlamydia* genome sequences should allow us to identify SNARE-like motifs in *Chlamydia* proteins with a high level of confidence. We restricted the search to proteins containing a large bilobed hydrophobic motif, which is the hallmark of Inc proteins [4]. These proteins are good candidates to interact with SNAREs, since they are most probably exposed on the cytosolic face of the inclusion membrane. Each *Chlamydia* genome encodes more than 40 putative Inc proteins. Among them, we found that the *C. trachomatis* genome encodes 11 proteins predicted to engage in coiled-coil interactions (see Materials and Methods). We aligned the 11 identified sequences against a SNARE motif profile compiled from 261 referenced SNAREs. The best score was obtained with a carboxy-terminal region of the inclusion protein IncA, which is anchored in the membrane by its N-terminal extremity (Figure 1A). We extended the search for SNARE motifs to all IncA sequences known to date, from a variety of *Chlamydia* strains. The level of similarity between IncA from different species is low, a characteristic of all Inc proteins [4]. Strikingly, in spite of this low conservation, SNARE-like motifs were identified in all IncA homologues, with the single exception of that of *C. pneumoniae* (Figure 1B). The conserved polar residue at the position corresponding to the zero layer of the SNARE motif was a glutamine or an arginine residue, which are the canonical residues in SNARE motifs. The presence of SNARE motif characteristics in IncA from six different *Chlamydia* strains strongly supports the hypothesis that the similarity to eukaryotic SNARE motifs is not fortuitous, and illustrates the usefulness of sequencing many strains of this intracellular bacterium which we can not genetically manipulate.

In addition to this motif, bioinformatics also revealed the presence of a second, membrane proximal, motif, which shared characteristics of SNARE motifs (Figure 1C). We designate it as motif Nter, and the carboxy-terminal motif as motif Cter. For each species, motif Cter always gave a higher score in the alignments with eukaryotic SNAREs than motif Nter. In particular, the polar residue in the zero layer of motif Nter was a threonine rather than a glutamine or an arginine for two *Chlamydia* species. Using a less systematic approach, we had previously observed that motif Nter showed similarities with SNARE motifs, and shown that it might be involved in the formation of homotetramers of IncA [15].

Finally, among the other *C. trachomatis* Inc proteins predicted to form coiled-coils, the Inc protein CT813 [16] and the putative Inc protein CT223 also showed similarities with SNARE motifs (amino acids 191 to 264 of CT813, 169 to 236 of CT223), although the alignment with host SNARE motifs was poorer than IncA's, and the zero layer was more difficult to define. Orthologs of CT813 and CT223 are only found in the closely related *C. muridiarium* species, so in this case sequence comparison could not be used to validate the identification of SNARE-like motifs. We did not identify SNARE-like motifs in the the eight remaining Inc proteins predicted to form coiled-coils.

### A subset of host SNAREs is specifically recruited to the inclusion membrane

**Figure 2:**
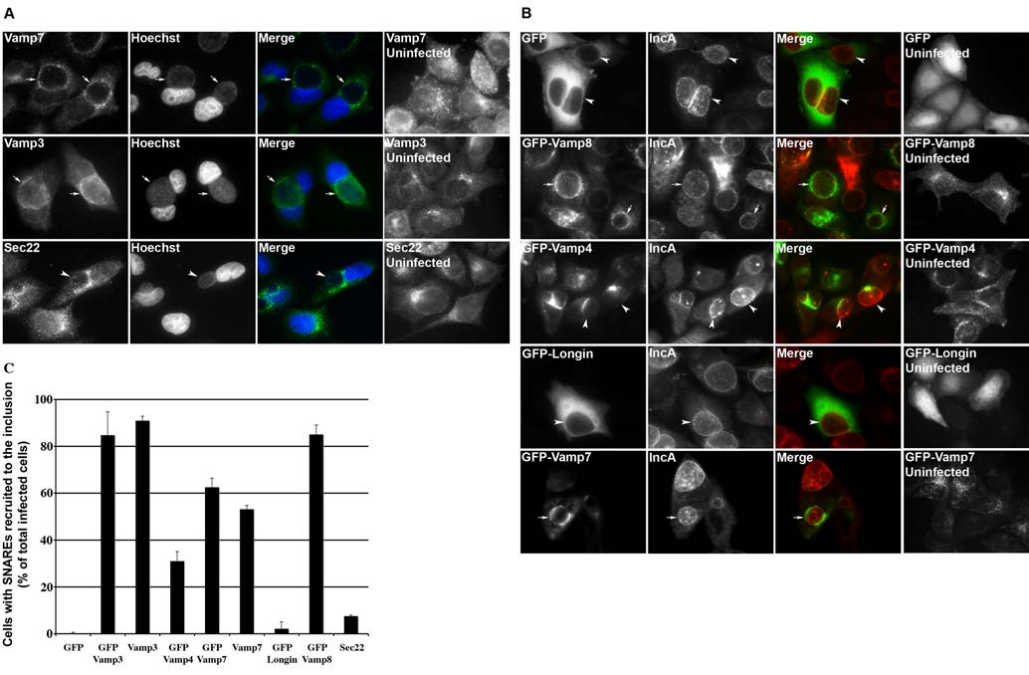
Several host SNARE proteins are specifically recruited around the inclusion membrane. A, Localization of endogenous SNAREs in HeLa cells infected for 24 h with *C. trachomatis* serovar D was assessed using specific antibodies. Vamp3 and Vamp7 encircle the inclusion (arrows), while Sec22 does not (arrowheads). SNAREs are shown on the left, host and bacterial DNA in the second column, and merged images in the third column. Distribution of the SNAREs in uninfected cells is shown on the right. B, Cells were transfected with different GFP-SNARE constructs 8 h before infection. The inclusion was labelled with anti-IncA antibodies followed with TRITC-coupled secondary antibodies. GFP-Vamp8 or GFP-Vamp7 encircle the inclusion membrane (arrows), while GFP, GFP-Vamp4 or GFP-Vamp7 deleted from its SNARE domain (GFP-Longin) do not (arrowheads). GFP-tagged SNAREs are shown on the left, IncA in the second column, and merged images in the third column. Distribution of the SNAREs in uninfected cells is shown on the right. C, Quantification of SNARE recruitment to the inclusion. Cells were scored as positive when the entire circumference of the inclusion was surrounded by the SNARE protein. Means and standard deviations of at least two independent experiments are shown.

To determine whether host SNAREs interact with inclusion proteins, we investigated the distribution of several of them in cells infected with *C. trachomatis* serovar D. Since *C. trachomatis* IncA SNARE-like motif Cter had a glutamine in its central layer, we hypothesized thas it might interact with R-SNAREs and investigated the localization of several host R-SNAREs, whose distribution and role in intracellular traffic is well documented (see Table 1 for details). In addition to their expected punctate distribution throughout the cell, endogenous Vamp3 and Vamp7 formed a patchy circle around the inclusion. Sec 22, another R-SNARE, did not encircle the inclusion, indicating that the redistribution observed in infected cells did not apply to all SNAREs (Figure 2A and C). Endogenous Vamp4 and Vamp8 expression was too low for detection. However, when cells were transfected with GFP-Vamp8 prior to infection, this protein showed a ring pattern around the inclusion, while GFP and GFP-Vamp4 did not (Figure 2B and C). Transfected GFP-Vamp7 relocalized to the inclusion while GFP-Vamp7 deleted of its SNARE motif (GFP-Longin) did not, indicating that the SNARE motif is necessary for the recruitment to the inclusion membrane.

**Table 1:** Distribution and known functions of host SNAREs used in this study.

SNAREs	Localization	Known Functions	References
**Vamp3**	RE	RE→TGN	[35]
		RE→PM	[36]
	EE	EE→PM	[37],[38]
**Vamp4**	TGN	TGN→Endosomes	[39]
	RE	EE/RE→TGN	[35],[40]
	EE	Homotypic fusion	[41]
	LD	Homotypic fusion	[42]
**Vamp7**	LE	LE→Lys	[43]
		LE→PM	[44]
	Lys	Lys→PM	[45]
**Vamp8**	EE	Homotypic fusion	[46]
	LE	Homotypic fusion	[47]
**Sec22**	ER	ER→Golgi	[48]
	Golgi	Golgi→ER	[49]

EE: early endosomes, ER: endoplasmic reticulum, LD: lipid droplets, LE: late endosomes, Lys: lysosomes, PM: plasma membrane, RE: recycling endosomes, TGN: *trans*-Glogi network.

**Figure 3:**
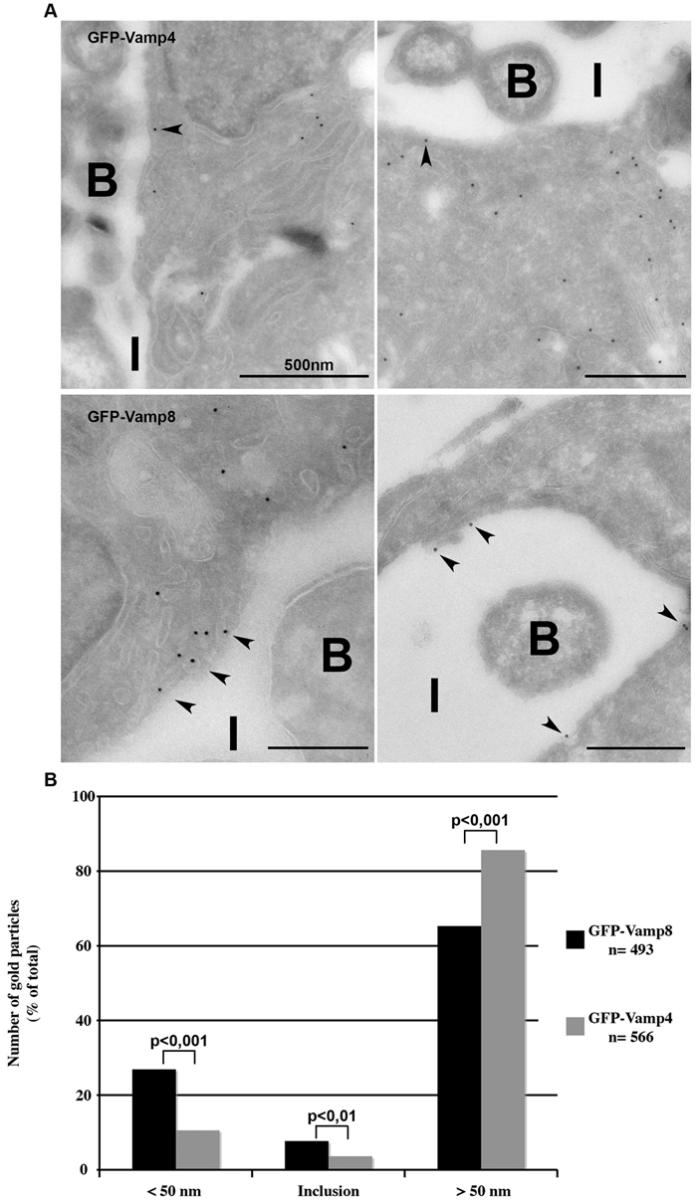
GFP-Vamp8 is located in compartments around the inclusion and in the inclusion membrane. A, Distribution of GFP was observed in fields with an inclusion, in cells transfected with GFP-Vamp4 (top) or GFP-Vamp8 (bottom), and infected. Two representative panels are shown for each construct. Arrowheads point to gold particles less than 50 nm from the inclusion membrane. I, inclusion, B, bacteria. B, Equivalent numbers of gold particles were counted in both sets of images and their localization relative to the inclusion membrane was assessed. Levels of significance are indicated by p-values comparing the distribution of GFP-Vamp4 and GFP-Vamp8 for each category.

The circular pattern of specific SNAREs can be due to their accumulation around the inclusion, and/or to their presence in the inclusion membrane itself. To discriminate between these possibilities, we used electron microscopy. In agreement with our observation that SNAREs of different compartments are recruited to the inclusion membrane, we observed an abundance of varied intracellular compartments in the immediate vicinity of the inclusion membrane (Figure 3A). Using immunogold labeling, we confirmed the enrichment of GFP-Vamp8 relative to GFP-Vamp4 around the inclusion, as 35% of the gold particles that labeled GFP-Vamp8 were less than 50 nm distant from its membrane, against 14% for those associated with GFP-Vamp4 (p-value<0.001). Among the gold particles that labeled GFP-Vamp8 within a 50 nm range of the inclusion membrane, 22% were on its membrane, while the rest were associated with compartments around the inclusion (Figure 3B). This result shows that the accumulation of GFP-Vamp8 around the inclusion observed in Figure 2B is mainly due to the recruitment of GFP-Vamp8 positive compartments around the inclusion, rather than to the presence of this host protein in the inclusion membrane. The same probably applies to GFP-Vamp3 and GFP-Vamp7 positive compartments, since endocytic markers of these compartments accumulate around the inclusion and are excluded from it [7]. Our observation that GFP-Vamp8 can reach the inclusion membrane, as well as the recent finding of CD63 on it, support the idea that multivesicular bodies, which contains both markers, might contribute to its growth [10].

### IncA interacts with host SNAREs

**Figure 4:**
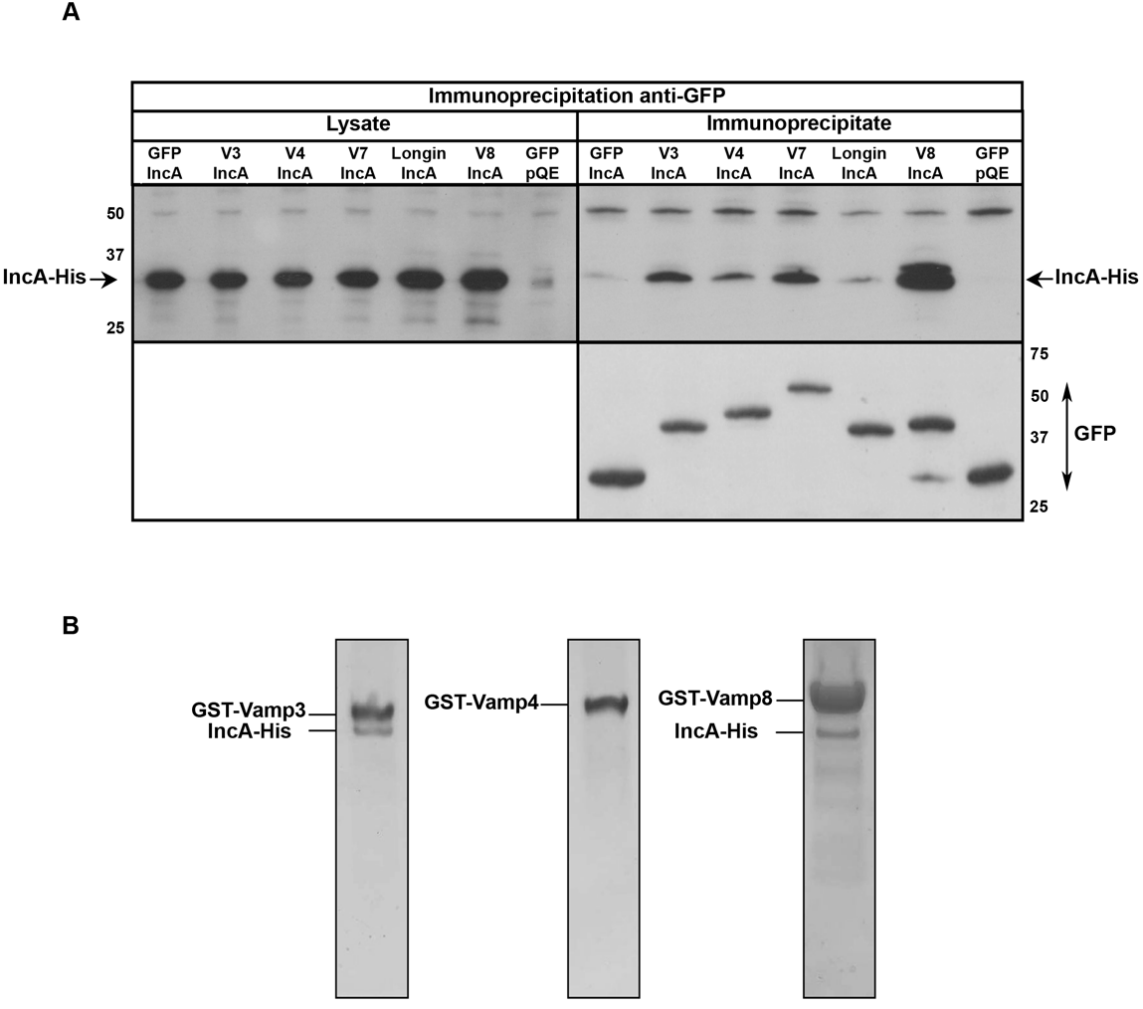
IncA interacts directly with host SNARE proteins. A, GFP-SNARE proteins (V3 = Vamp3, V4 = Vamp4, V7 = Vamp7, V8 = Vamp8, Longin = Vamp7 without its SNARE motif) were immunoprecipitated from HeLa cells co-expressing IncA or empty vector (pQE). The top left panel (anti-His blot) shows the level of expression of IncA in cell lysates, and the bottom right panel (anti-GFP blot) shows immunoprecipitation of GFP-tagged proteins. IncA co-immunoprecipitated with Vamp3, Vamp7 and Vamp8, and very little with Vamp4 (top right panel, anti-IncA blot). B, Purified IncA-His and purified GST-Vamp3, GST-Vamp4 or GST-Vamp8 were inserted in two populations of liposomes, mixed, and GST-SNAREs were pulled-down using glutathione agarose beads. Pull-down complexes were resolved on SDS-PAGE gels and analyzed after staining with Coomassie.

The recruitment of Vamp8 positive compartments in the immediate proximity of the inclusion membrane suggests a possible direct interaction between this SNARE and SNARE-like proteins on the inclusion membrane. We next asked whether IncA could mediate the recruitment of Vamp8-positive compartments, and of other intracellular compartments. HeLa cells were simultaneously transfected with plasmids coding for *C. trachomatis* IncA and with different GFP-tagged SNARE proteins. One day later, SNARE proteins were immunoprecipitated using anti-GFP antibody. IncA was present in the GFP-immunoprecipitate when co-expressed with GFP-Vamp3, GFP-Vamp7 or GFP-Vamp8, but not GFP alone (Figure 4A). Importantly, IncA was not found in the GFP-immunoprecipitate when co-expressed with GFP-Vamp7 deleted of its SNARE motif (GFP-Longin), indicating that the SNARE motif of the SNARE protein is important for the interaction with IncA. It co-immunoprecipitates very poorly with GFP-Vamp4, indicating that IncA interacts preferentially with only a subset of SNAREs. These biochemical observations correlate well with the selective recruitment of SNAREs around the inclusion observed in Figure 2.

*In vitro* pull-down assays were performed to determine whether the interaction between IncA and host SNAREs was direct. Two populations of liposomes, one containing purified Vamp3, Vamp4 or Vamp8 with an amino-terminal glutathione-S-transferase tag, and one containing purified IncA-His, were mixed and incubated together for 16 hrs at 4°C. Proteins were then solubilized, and Vamps were pulled-down using glutathione-agarose beads (Figure 4B). IncA was pulled-down together with Vamp3 or Vamp8, showing that the interaction between IncA and these SNAREs is direct. IncA was not pulled-down with Vamp4, which correlates with the very weak co-immunoprecipitation of this inclusion protein with GFP-Vamp4 observed in Figure 4A, and confirms that IncA interacts preferentially with only a subset of SNAREs.

### IncA plays a predominant role in the interaction with SNAREs

IncA is not expressed by *C. trachomatis* until 10 hrs post-infection [17]. To see whether the timing of the recruitment of SNAREs was consistent with the temporal expression of IncA, we observed the localization of SNAREs early in infection. Eight hours after infection, inclusions were very small, and we could not quantify the recruitment of SNAREs with confidence at this stage (data not shown). Eleven hours after infection, the recruitment of GFP-SNAREs was much less pronounced than 18h after infection (Figure S1), showing that the level of recruitment of SNAREs correlates with the timing of expression of IncA.

**Figure 5:**
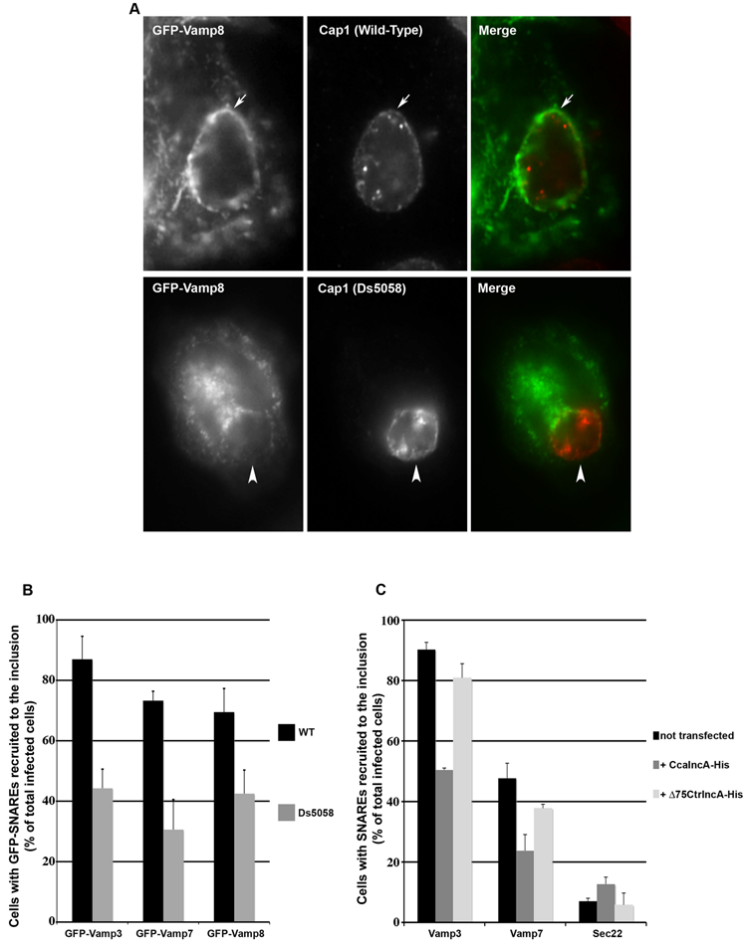
IncA plays a predominant role in SNAREs recruitment to the inclusion. A, The recruitment of transfected GFP-Vamp8 (left) around the inclusion membrane (labelled with antibodies against the inclusion protein Cap1, middle) is reduced in cells infected with a IncA defective mutant strain (Ds5058, bottom panels) compared to wild type (top panels). B, The percentage of cells (n>100) in which the indicated GFP-SNARE proteins encircle the inclusions is reduced in cells infected with the IncA defective mutant strain (grey) compared to wild type (black). Coverslips were analyzed in duplicate by different investigators, and the means and standard deviations of the two resulting counts are shown. C, The percentage of cells (n>100) in which the indicated endogenous SNAREs encircle the inclusions in control cells (black), cells overexpresing *C. caviae* IncA (dark grey) or *Δ*75CtrIncA (light grey) is shown. The means and standard deviations of two experiments are shown.

To test directly IncA's contribution to the recruitment of SNAREs to the inclusion membrane, we used a strain of *C. trachomatis* that does not express IncA, Ds5058 [18]. This strain grows significantly slower than the wild type strain [19], indicating that IncA plays an important role in infection. Cells were transfected with GFP-Vamp8, and then infected with the IncA negative strain. The cells were incubated for 48 hrs, in contrast to 24 hrs for the wild type strain, in order to observe inclusions of similar size (Figure 5A). Recruitment of GFP-Vamp8 to the inclusion was observed in about 40% of cells infected with the IncA negative strain, in contrast to 70% of cells infected with the wild type strain. Similarly, the circular pattern of GFP-Vamp3 or GFP-Vamp7 around the inclusion was observed in fewer cells infected with the IncA negative strain than with wild-type bacteria. At this level of analysis, in cases where host SNAREs were recruited to the IncA negative inclusions, no difference could be noted with the pattern of their distribution in cells infected with the wild type strain (Figure 5B and Figure S2).

We used an independent approach to confirm the predominant role of IncA in SNARE recruitment. As IncA associates with itself [15], we reasoned that heterologous overexpression of IncA might titrate the protein on the inclusion membrane and prevent association with SNAREs. Unfortunately, overexpression of *C. trachomatis* IncA inhibits the development of the bacteria [15], and could not be used for this purpose. However, we had previously shown that IncA from *C. trachomatis* (CtrIncA) and *C. caviae* (CcaIncA) shared biochemical properties [15] and we have identified here two SNARE-like motifs in *C. caviae* IncA (Figure 1B and C). We hypothesized that CcaIncA, if expressed by the infected cell, might associate with endogenous CtrIncA at the inclusion membrane. Indeed, when overexpressed by HeLa cells, CcaIncA was present in the endoplasmic reticulum, as previously observed [15], and around the inclusion membrane (Figure S3). The number of cells in which Vamp3 and Vamp7 were scored as recruited to the inclusion was reduced by about 50% in the population of cells expressing CcaIncA compared to non transfected cells (Figure 5C and Figure S3). Interestingly, overexpression of CtrIncA deleted from its hydrophobic domain (*Δ*75IncA) was not enriched around the inclusion and had no effect on the recruitment of Vamp3 and Vamp7 to the inclusion (Figure 5C). This result confirms our previous observation that overexpressed IncA needs to be inserted into a membrane compartment to be able to interact with endogenous IncA [15].

### Other inclusion proteins might interact with the SNARE machinery

**Figure 6:**
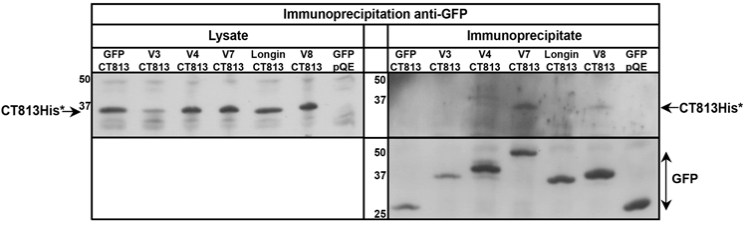
CT813 can interact with host SNAREs. GFP-SNARE proteins (V3 = Vamp3, V4 = Vamp4, V7 = Vamp7, V8 = Vamp8, Longin = Vamp7 without its SNARE motif) were immunoprecipitated from HeLa cells co-expressing CT813-His* or empty vector (pQE). The top left panel (anti-His blot) shows the level of expression of CT813-His* in cell lysates, and the bottom right panel (anti-GFP blot) shows immunoprecipitation of GFP-tagged proteins. CT813-His* co-immunoprecipitated with Vamp7 and Vamp8 (top right panel, anti-His blot), and not with Vamp3, Vamp4, GFP or GFP-Longin. Absence of immunoprecipitation with Vamp3 might be due to insufficient expression level of both CT813-His* and GFP-Vamp3, which were consistently low in these experiments.

These results indicate that IncA plays a predominant, although not exclusive, role in the recruitment of SNAREs around the inclusion. Other inclusion proteins such as CT223 and CT813, which show similarities with SNARE motifs, may contribute to SNARE recruitment around the inclusion. To test this hypothesis, we cloned these two genes in mammalian expression vectors. After transfection, CT223 was not expressed by HeLa cells, as assessed by immunofluorescence, and was not studied further. CT813 was expressed in the endoplasmic reticulum. Co-expression and immunoprecipitation experiments showed that CT813 interacts with GFP-Vamp7 and GFP-Vamp8, and not with GFP alone GFP-Vamp4 or GFP-Vamp7 deleted of its SNARE motif (GFP-Longin). Interaction of CT813 with GFP-Vamp3 could not be assessed because the expression levels of both constructs were low (Figure 6). This result indicates that CT813 can interact with host SNAREs and that, in addition to IncA, several Inc proteins have evolved the ability to interact with SNAREs. Interestingly, more CT813 was pulled-down with Vamp7 than with Vamp8, while more IncA was pulled down with Vamp8 than with Vamp7, suggesting that affinities between inclusion proteins and different SNAREs vary. Other Inc proteins might partly compensate for the absence of IncA and explain why the clinical strain defective in IncA expression was still able to recruit SNAREs around its inclusion, although less efficiently that the wild type strain. Interestingly, CT813 and CT223 are specific to *C. trachomatis* and *C. muridarium*, suggesting that different *Chlamydia* species may have evolved different Inc proteins, targeting the SNARE machinery of their host in subtly different manners. In support of this hypothesis, we observed that, while *C. caviae* and *C. pneumoniae* were also able to recruit SNAREs to the inclusion membrane, the level of recruitment varied with strains (Figure S4). *C. caviae*, whose IncA has SNARE-like motifs and behaves similarily to *C. trachomatis* IncA [15], recruited the same set of SNAREs as *C. trachomatis*. In contrast, *C. pneumoniae*, whose IncA does not possess a clear SNARE-like motif, recruited good level of Vamp8 in the vicinity of its inclusion, but only little Vamp7. This observation suggests that, in *C. pneumoniae*, other Inc proteins than IncA might specifically interact with a subset of SNAREs.

Importantly, targeting the host SNARE machinery is not the sole method for Inc proteins to interfere with host trafficking: members of the family of rab proteins, which also participate in recognition and fusion of cell compartments [20], were also shown to interact with Inc proteins [21],[22].

### IncA SNARE-like motifs fit in the structure of the SNARE complex and function as bona fide SNARE motifs

**Figure 7:**
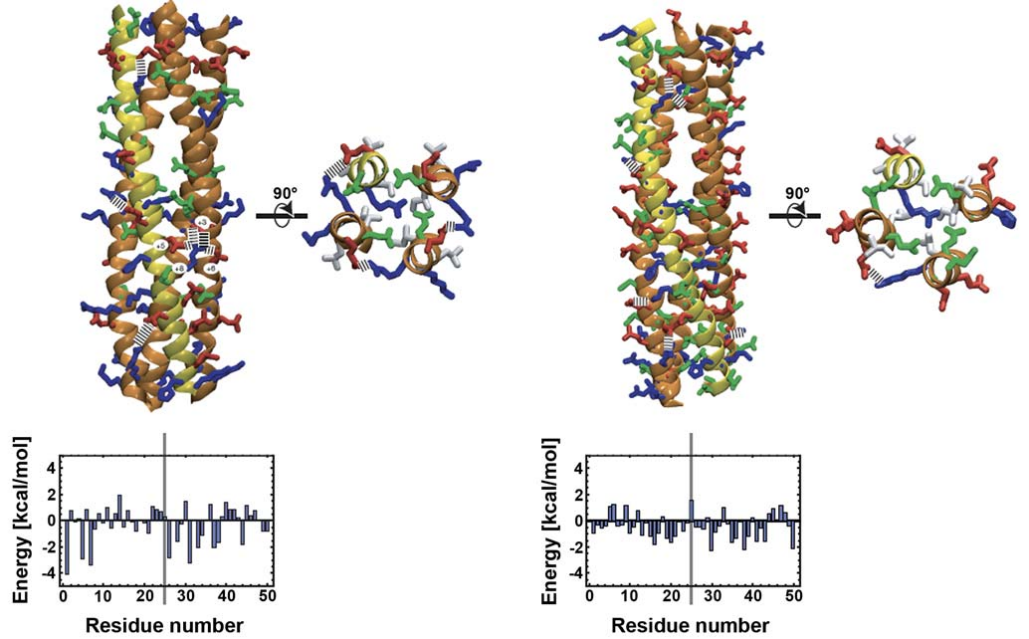
IncA SNARE-like motifs fit in the structure of the SNARE complex. Side view of the complex of three motifs Cter from *C. trachomatis* IncA with the SNARE motif of Vamp8 (left) and of the endosomal SNARE complex [23] (right), with top view of the central layer for both complexes. The backbone of the helices is shown in orange for IncA and yellow for Vamp8. Dashed lines indicate favorable electrostatic interactions. Acidic residues (Asp, Glu) are shown in red, basic residues (Arg, Lys, His) in blue, and other polar residues (Asn, Glu) in green; in the top views hydrophobic residues are shown in white, and all other residues are left out for clarity. The energetic profiles at the bottom are estimates of the total contribution of a residue to the stability of the complex, averaged over the four helices (see Materials and Methods). The vertical line indicates the position of the central layer. Negative energy indicates that a residue is predicted to stabilize the complex, positive energy that it destabilizes the complex.

Using molecular modelling, we previously showed that motif Nter was fully compatible with the formation of stable homotetramers, associated in a structure similar to that of the SNARE complex [15]. Using the same approach, we now asked whether IncA SNARE-like motifs could fit in the structure of a SNARE complex involving host SNAREs. We chose to use IncA SNARE-like motif Cter because it aligned better with eukaryotic SNARE motifs than motif Nter (Figure 1). We modelled the association of three identical motifs Cter (in place of Q-SNAREs) in association with one SNARE motif from a R-SNARE, for three reasons: (i) IncA has a high propensity to form dimers or other multimeric structures [15], (ii) motif Cter of *C. trachomatis* IncA classifies as a Q-SNARE, and aligned better with Q-SNAREs than R-SNAREs (Figure 1B) (iii) IncA can interact with R-SNAREs (Figure 4). We modelled the heterotetramers between the SNARE motif of host Vamp8 and a trimer of IncA motif Cter (Figure S5). The model of the complex between three molecules of IncA and Vamp8 is very similar to the structure of the endosomal SNARE complex (Figure 7). In particular, several side-chains of Vamp8 are involved in salt bridges with side-chains in IncA. There is a cluster of salt bridges close to the central layer of the complex, involving residues at positions +5 and +8 from the central Argnine in Vamp8, with resdidues at positions +3 and +6 from the central Glutamine in IncA, and two additional salt bridges N-terminal and C-terminal of the central layer. The patterns of predicted interaction energies, evaluated as the difference in total energy between the complex and separated helices, are similar. The predicted arrangement of glutamine residues around the central arginine of Vamp8 is very similar to that found in the X-ray crystal structure of the endosomal SNARE complex [23] (Figure 7, inset). These data support a model in which IncA makes SNARE complexes with host SNAREs via its SNARE-like motif Cter.

**Figure 8:**
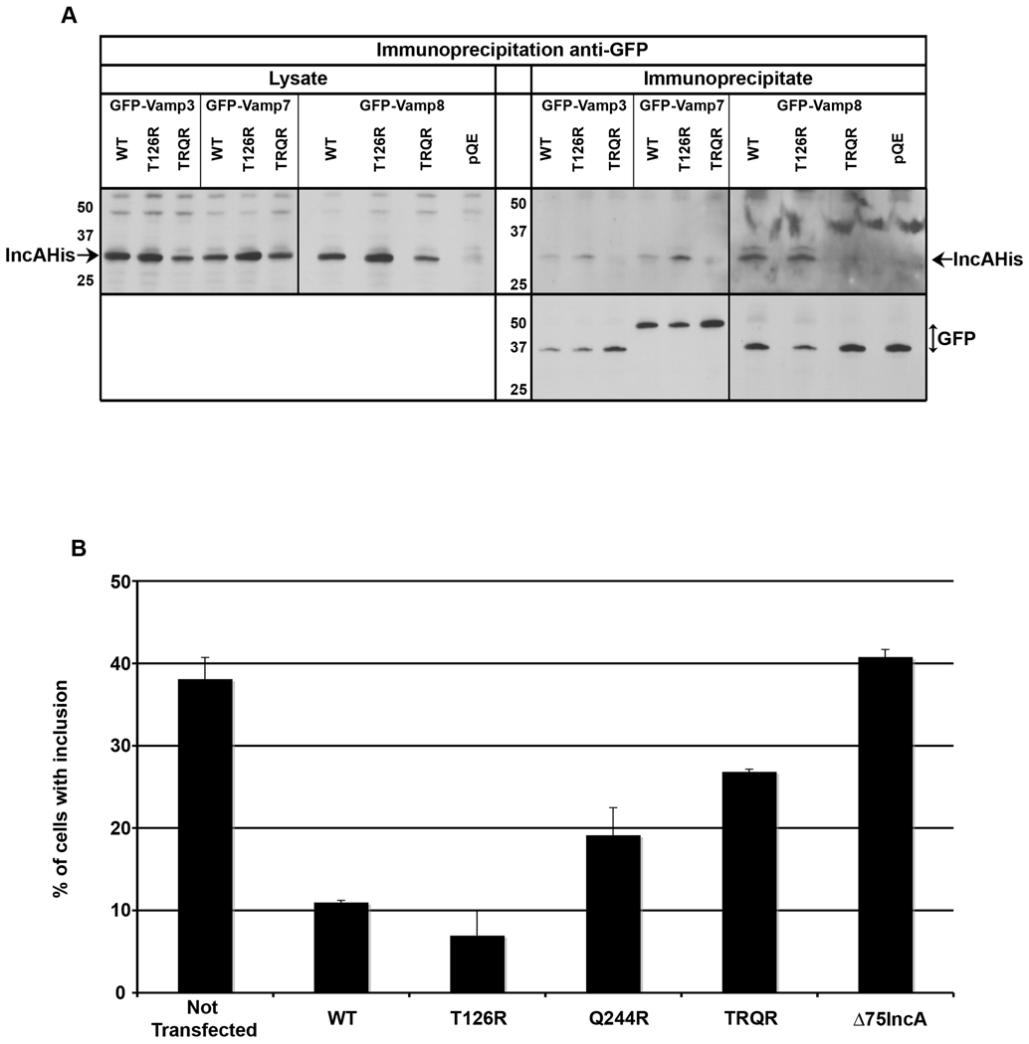
Effect of point mutations in the central layer of IncA SNARE-like motifs. A, GFP-SNARE proteins were immunoprecipitated from HeLa cells co-expressing IncA wild-type, mutated in motif Nter (T126R) or in both SNARE-like motifs (TRQR), or empty vector (pQE) as control. The top left panel (anti-IncA blot) shows the level of expression of IncA in cell lysates, and the bottom right panel (anti-GFP blot) shows immunoprecipitation of GFP-tagged proteins. IncA wild-type and T126R immunopecipitated with the SNAREs, and the double mutant did not (anti-IncA blot,top right panel). B, Cells transfected for 18 hrs with the indicated His-tagged IncA constructs were infected for 20 h with *C. trachomatis* serovar D before fixation. Coverslips were stained with anti-His antibodies and anti-EfTu antibodies to label the bacteria. Histograms show the percentage of cells (n>100) with a normal inclusion for each population of transfected cells, in one experiment representative of three (means and standard deviations of two independent counts are shown). Only inclusions that looked intact were scored; in cells transfected with IncA wild-type and IncAT126R, and, to a lesser extent, IncAQ244R and IncAT126RQ244R (TRQR), many disrupted inclusions were also observed.

Our models, and numerous reports on eukaryotic SNARE complexes, suggest that, if the IncA SNARE-like motif functions as a bona fide SNARE motif, forming complexes with R-SNAREs, introduction of a large charged amino acid, such as an arginine, in the zero layer of the IncA SNARE-like motif might destabilize the SNARE complex sufficiently to lose the interaction between IncA and host SNAREs. To test this hypothesis, we introduced point mutations in the zero layer of IncA SNARE-like motif Nter (IncAT126R), motif Cter (IncAQ244R) or both (IncAT126RQ244R). All constructs were expressed at a level comparable to wild type IncA when transfected in HeLa cells. However, coexpression of the Q244R mutant together with GFP-Vamps constructs resulted in very weak expression of both transfected genes. The reasons for this phenomenon are unclear, and we could not assay the ability of this mutant to co-immunoprecipitate with GFP-SNAREs. We performed co-immunoprecipitation experiments in cells co-expressing different GFP-SNAREs and either IncA wild type, or mutated in motif Nter, or in both SNARE-like motifs. The single mutant (IncAT126R) co-immunoprecipitated with GFP-Vamp3, GFP-Vamp7 and GFP-Vamp8, to the same extent as IncA wild-type. However, IncA mutated in both SNARE-like motifs (IncAT126RQ244R) did not co-immunoprecipitate with any of the Vamps (Figure 8A). This experiment demonstrates that the interaction between IncA and host SNAREs is mediated by IncA SNARE-like motifs. Further analysis would be needed to determine whether only motif Cter is able to engage in SNARE complexes with host SNAREs, or whether any of the two SNARE-like motifs can do so and mutation of both motifs is needed to lose the interaction. In any case, these experiments demonstrate that IncA SNARE-like motifs function as bona fide SNARE motifs since point mutations in residues known to be critical for the formation of SNARE complexes result in the loss of the interaction between IncA and host SNAREs.

To confirm this important result by a different approach, we investigated the effect of the point mutations in IncA SNARE-like motif on the ability of IncA to engage in homotypic interactions. We have previously shown that heterologous expression of IncA, which localizes at the endoplasmic reticulum, inhibits inclusion development. This effect requires a direct interaction between IncA molecules at the inclusion and on the endoplasmic reticulum, and we hypothesized that it might be due to the formation of homotypic SNARE complexes between IncA molecules present on the two compartments [15]. Cells, transfected with IncA wild- type or with the different mutants, were infected for 20 hrs before fixation and labeling of the bacteria and IncA-His by immunofluorescence. Development of inclusions was largely impaired in cells expressing IncA wild type or IncAT126R (Figure 8B). The Q244R mutation in the IncA SNARE-like motif Cter partially restored the growth of the inclusion in the transfected population, and the double mutation in both SNARE-like motifs restored the growth further, although not totally. Expression of IncA deleted of its hydrophobic domain (Δ75IncA) had no effect on the development of the bacteria, confirming that IncA needs to be inserted in a membrane to disrupt the development of the inclusion [15]. Altogether, this experiment shows that homotypic interaction between IncA molecules is mediated by its SNARE-like motifs. It suggests that both SNARE-like motifs contribute to the interaction, although motif Cter is able to compensate for the mutation in motif Nter, while motif Nter is not fully able to compensate for the mutation in motif Cter. The observation that the double mutant only partially (about 50%) restores the ability of the bacteria to grow in the transfected cells indicates that the point mutations are not sufficient to fully disrupt IncA homotypic interactions.

Altogether, point mutations in IncA SNARE-like motifs impaired its ability to associate with host SNAREs and with itself. These results show that IncA SNARE-like motifs behave as bona fide SNARE motifs, and strongly support our hypothesis that IncA interaction with host SNAREs is mediated by the formation of SNARE complexes.

### How does IncA affect membrane traffic?

In agreement with previous reports from several laboratories, we have observed that a variety of cellular compartments accumulate around the inclusion. Here we bring evidence that SNARE mimicry is one mechanism by which the *Chlamydia* recruit a specific subset of host SNAREs. We have identified SNARE-like motifs in the inclusion protein IncA, and showed that a mutant strain that does not express IncA presents reduced ability to recruit SNAREs around its inclusions. Our data also suggest that other inclusion proteins may use SNARE motif mimicry to interact with SNAREs. These conclusions open two important questions: in which SNARE complexes is IncA engaged, and what are the consequences in term of membrane fusion?

To start answering the first question, we have tested a variety of IncA/SNARE interactions. By co-imunoprecipitation experiments, we found that IncA was able to interact with several different SNAREs, but not all. We observed a good correlation between this result and the specific recruitment of a subset of SNAREs around the inclusion membrane. The basis of the specificity of interaction between IncA and a subset of host SNAREs is not yet known. A model between the SNARE motif of Sec22 and a trimer of IncA motif Cter resulted in an unstable complex (data not shown), a finding that correlates with the absence of recruitment of Sec22 around the inclusion membrane. However, modelling predicted a stable association of Vamp4 with IncA motif Cter, while we observed no or very weak interaction between IncA and Vamp4 (Figure 4). This result suggests that, in addition to motif Cter, other domains of IncA contribute to define its specificity. It may also be that, *in vivo*, interactions between the inclusion membrane and the host SNAREs involve a combination of SNARE-like motifs from several different inclusion proteins and/or of SNARE motifs from several different host SNAREs present in the same compartment. Dissecting the complexes in which IncA is involved therefore remains a challenging task for the future.

It is also difficult to bring a definite answer to the second question, regarding the mechanistic roles of SNARE-like motifs in inclusion proteins, especially in the absence of tools to manipulate the *Chlamydia* genome. The three SNAREs recruited to *C. trachomatis* inclusion membrane are SNAREs involved in endosomal trafficking, while Vamp4 and Sec22, which are more involved in the secretory pathway, were not, suggesting that SNARE mimicry may have preferentially evolved in *C. trachomatis* to target the endosomal pathway. Depletion of host SNAREs Vamp3, Vamp4, Vamp7 or Vamp8 using siRNA had no impact on the growth of the bacteria (Figure S6). This might be due to an insufficient depletion of the pools, especially in the case of Vamp3, or because other host SNAREs present in the same compartment as the siRNA target can compensate for the depletion. It might also reveal some degree of redundancy in the interactions between the inclusion and different intracellular pathways, as was observed for another intracellular bacterium, *Legionella pneumophila* [24].

In the absence of functional indications regarding the role of the interaction between inclusion proteins and host SNAREs in infection, we can propose several models. SNAREs are mostly known for their role in membrane fusion. By engaging in fusion competent SNARE complexes, inclusion proteins might permit the fusion of some cellular compartments with the inclusion membrane. If this is the case, the cellular SNARE should be present in the inclusion membrane, at least transiently. By electron microscopy, we observed that Vamp8 is found in the inclusion membrane, suggesting that indeed, IncA-SNARE interaction might promote fusion of specific intracellular compartments, in this case probably multivesicular bodies, with the inclusion. Conversely, SNARE motifs in inclusion proteins might play an inhibitory role on fusion, by engaging in fusion incompetent SNARE complexes [13], or by titrating individual SNAREs. One argument to support this opposite model is the long distance between motif Cter and the transmembrane domain of IncA. SNARE mediated fusion requires proximity between the SNARE complex and the lipid bilayers, which might be difficult to achieve if motif Cter is engaged in the SNARE complex. Another observation that supports this model is the absence of effect of Vamp7 depletion on infection. Although siRNA against Vamp7 reduced its level by more than 90%, it did not affect the growth of the bacteria (Figure S6). Similarily, expression of GFP-Longin, a dominant negative form of Vamp7, did not affect *Chlamydia* infection. These results support a model in which IncA/Vamp7 interaction prevents Vamp7 mediated fusion of late endocytic compartments with the inclusion. Finally, it is also possible that SNARE-like domains in inclusion proteins serve as mere anchors. By associating with various SNAREs, they contribute to the accumulation of vesicles in the immediate vicinity of the inclusion. This might be beneficial to the development of the bacteria, without requiring fusion to occur.

*Chlamydiaceae* have very probably been intracellular for several hundred million years [25]. Therefore, a high degree of complexity in the interaction between inclusion proteins and host organelles should be expected. Bacterial proteins involved in these interactions are just beginning to be identified. In light of our results, we propose that co-evolution shaped some of the bacterial inclusion proteins into bacterial SNARE proteins.

## Materials and Methods

### Infection and immunofluorescence microscopy

*C. trachomatis* serotype D (27F0734) was from ATCC. *C. trachomatis* serotype D(s)5058 is a clinical isolate, which does not express IncA, and was kindly given by Drs. D. Rockey and W. Stamm [18]. The GPIC strain of *C. caviae* and the CWL029 strain from *C. pneumoniae* were obtained from Drs. R. Rank (University of Arkansas) and G. Christiansen (University of Aarhus, Denmark) respectively. HeLa cells were used in all experiments except for infection with *C. pneumoniae*, which was performed in Hep2 cells. Infections were performed as described [15]. In some experiments, HeLa cells were transfected 8 to 18 hrs prior to infection and processed for immunofluorescence as described [15]. The inclusion membrane was observed using either anti-IncA (generous gift from Dr Ted Hackstadt, Rockey Mountain Laboratories, NIH, NIAID) or anti-Cap1 polyclonal antibodies which we obtained from New Zealand White rabbits immunized with His-tagged recombinant protein CT529 purified from *E. coli*. Antibodies against Vamp4 were kindly provided by Dr. Andrew A. Peden (Cambridge Institute for Medical Research, Cambridge), rabbit anti-Vamp3, rabbit anti-Vamp8 and mouse anti-Vamp7 and vectors coding for GFP-Vamp3, GFP-Vamp7, GFP-Longin and GFP-Vamp8 were kindly provided by Dr Thierry Galli (Institut Jacques Monod, Paris), rabbit anti-Sec22 was described previously [26], plasmid coding for GFP-Vamp4 was a gift of Dr Ludger Johannes (Institut Curie, Paris). Endogenous SNAREs were first labeled using the corresponding antibodies, followed with Alexa Fluor-488-conjugated anti-rabbit antibodies (Molecular Probes). In that case, the inclusion membrane could not be visualized with rabbit antibodies, and bacterial DNA was labeled using 0.5 µg/ml Hoechst in the mounting medium. Coverslips were examined under an epifluorescence microscope (Axiophot, Zeiss, Germany) equipped with a 63× Apochromat objective and a cooled CCD-camera (Photometrics, Tucson, AZ), driven by Metaview software (Universal Imaging, Downingtown, PA). For quantification of the recruitment of SNAREs around the inclusion membrane, more than 100 infected cells (and in some case transfected with GFP-SNARE) were counted in each case. Cells were scored as positive when the entire circumference of the inclusion was surrounded by the SNARE protein.

### Immunoprecipitation assay

HeLa cells were transfected by electroporation with the indicated constructs, and lysed 24 h later on ice in 50 mM Tris, 150 mM NaCl, 1% Triton X-100, 10 mM EDTA, pH 7.5 and cocktails of inhibitors. Immunoprecipitation of GFP-tagged proteins was performed using anti-GFP monoclonal antibodies (clones 7.1 and 13.1, Roche Applied Science). Immunoprecipitated proteins were incubated in sample buffer, boiled and loaded on acrylamide gel for western blot detection of IncA or of the histidine tag (#14-6757, eBioscience, San Diego CA). Expression level and immunoprecipitation of SNAREs proteins were checked by stripping the membrane and incubating it with anti-GFP antibodies (#sc-8334 Santa Cruz Biotechnology) followed by horseradish peroxidase-linked (HRP) anti-rabbit antibodies (Amersham Biosciences).

### Pull-down experiments

GST-Vamp3, GST-Vamp4, and GST-Vamp8 were expressed, purified and reconstituted into a first population of clear liposomes as described [27],[28]. Full-length IncA-His was subcloned in the pet28 vector, expressed in BL21 *E. coli* for 20 hrs at 16°C, and subsequently purified in buffer A (100 mM KCL, 25 mM HEPES, 10% glycerol, 1% octyl-β-D-glucopyranoside) using the protocol described in [27]. IncA-His was reconstituted into a second population of clear liposome as described [28]. After incubating each GST-SNAREs containing liposome with IncAHis-containing liposomes together for 16 hrs at 4°C, the mixture was dissolved in 2.5% wt/vol n-dodecyl-maltoside (Boehringer). The proteins/lipids mixture was further diluted into buffer A, and GST complexes were pulled-down using glutathione agarose equilibrated in buffer A. The glutathion agarose beads were then pelleted and washed three times with the buffer A. Pull-down complexes were resolved on SDS-PAGE gels and proteins stained with Coomassie blue.

### Electron microscopy

Transfected and infected HeLa cells were fixed with a mixture of 2% (wt/vol) paraformaldehyde and 0.5% (wt/vol) glutaraldehyde in a 0.2 M phosphate buffer (PB) pH 7.4, 4 h at room temperature. After many washing with 0.1 M glycin in PBS, cells were processed for ultracryomicrotomy as described [29]. Incubations were performed in blocking buffer (20 mM glycin, 0.1% of cold water fish skin gelatin, Biovalley, in PBS) and sections were labeled with anti-GFP (#A11122, Invitrogen Life Technologies) and visualized with protein A coupled to 15-nm gold. Sections were observed and acquired under a Philips CM120 electron microscope (FEI, Eindoven, Netherlands). Digital acquisitions were made with a numeric camera Keen View (Soft Imaging System, Munster, Germany).

### Expression of inclusion proteins

Construction of CtrIncA and CcaIncA with a carboxyterminal His tag was described elsewhere [15], Δ75IncA-His was constructed in the same way with a deletion of the first 75 amino acids. The same cloning strategy was used to clone CT813 using the primers atgctaccatggctactcttcccaataattgcactt and agtcggtacctatcgaaccacgtcttcctg, but for unknown reasons, the gene corresponding to the full-length protein could not be cloned over several attempts. A clone, designated CT813His*, was obtained, with a deletion of one nucleotide which did not permit expression of the full-length protein, but of a protein missing its 23 first amino acids (initiation of translation from methionine 24). This deletion does not affect the transmembrane domain of CT813, or its SNARE-like motif.

### Depletion of SNAREs by siRNA

The selection of specific oligonucleotides and the procedures for RNAi experiments to silence the expression of endosomal v-SNAREs (Vamp3, Vamp7, Vamp8) will be published elsewhere (Danglot et al., 2008 in preparation), Vamp4 siRNA (AAGAUUUGGACCUAGAAAUGA) was designed by Dr. Andrew A. Peden (Cambridge Institute for Medical Research, Cambridge). 5×10^6^ cells were electroporated in the presence of 2 pmoles of siRNA at day 1 and again at day 4, plated in 24-well plates and one 10-cm dish at day 5 and infected at day 6 in the 24-well plates. On the day of infection, cell lysates were prepared from the 10 cm dish and normalized to equal protein concentration. The level of expression of each SNARE was assessed by western blot using specific antibodies. Nitrocellulose membrane were incubated with ECL western blotting reagents (Amersham) and processed for detection using a Luminescent Image Analyzer LAS-3000 (Fujifilm). Digital images were acquired using the software Image Reader LAS-3000 v2.2 (Fujifilm) and analyzed by MultiGauge v3.0 (Fujifilm). All the quantification were performed using the expression of β-tubulin to normalize the samples, and for each SNARE, the level of expression is expressed as the percentage of expression in control cells transfected with the si-β-globin. One day after infection, the cells were detached from 24-well plates in 0.5 mM EDTA in PBS, fixed for one hour in 70% ethanol, centrifuged and washed in PBS. Bacteria were then stained using anti-EfTu antibody from Dr Y-X Zhang (Boston) followed by Cy5-coupled anti-mouse antibody (Amersham) and the samples were analyzed by flow cytometry.

### Sequence analysis

Transmembrane domains were identified in *Chlamydia* proteome using Phobius predictor, which detects in a single search signal peptides and transmembrane helixes [30]. Proteins with two transmembrane domains separated by 2 to 40 amino acids, and with no obvious functional attribution, were considered as putative Inc proteins. Putative Inc proteins predicted to engage in coiled coil using a hidden markov model (Marcoil [31]) were CT119 (IncA), CT222, CT223, CT224, CT225, CT226, CT228, CT229, CT233 (IncC), CT616 and CT813. A database made of SNARE motifs from 261 referenced SNARE proteins was used to perform optimal alignments searches [32].

### Structural and energetic analysis

Models of the complexes were generated essentially as described before [15], see Figure S5. Electrostatic energies were calculated with the “ACE” Generalized Born model [33], implemented in X-plor [34], with an dielectric constant of 1 for the interior of the protein, and a cut-off for electrostatic interactions of 15 Å. To estimate the relative stabilities of the complexes, we calculated the difference between the energies for the tetramer and the four helices separated by 200 Å. Solvatation energies were estimated by the difference in solvent accessible surfaces multiplied by 0.005 kcal/(mol Å2). The profiles shown in Figure 6 represent the sum of electrostatic energies, van der Waals energies, and solvatation energies, averaged over the equivalent residues in the four helices.

## Supporting Information

Figure S1 The level of recruitment of SNAREs to the inclusion correlates with the timing of expression of IncA.
(2.00 MB TIF)

Figure S2 IncA is involved in the recruitment of Vamp3 and Vamp7 to the inclusion.
(8.90 MB TIF)

Figure S3 Expression of *C. caviae* IncA inhibits the recruitment of Vamp3 and Vamp7 to *C. trachomatis* inclusions.
(9.99 MB TIF)

Figure S4 Recruitment of GFP-SNAREs around *C. caviae* and *C. pneumoniae* inclusions.
(1.91 MB TIF)

Figure S5 Modelling.
(6.23 MB TIF)

Figure S6 Depletion of Vamp3, Vamp4, Vamp7 or Vamp8 does not affect the development of *C. trachomatis*.
(2.63 MB TIF)
