# Supplementary figures and images for: SNARE Protein Mimicry by an Intracellular Bacterium
Source: PLoS Pathog. 2008 Mar 14;4(3):e1000022. doi: 10.1371/journal.ppat.1000022 (PMC2265411; doi:10.1371/journal.ppat.1000022)

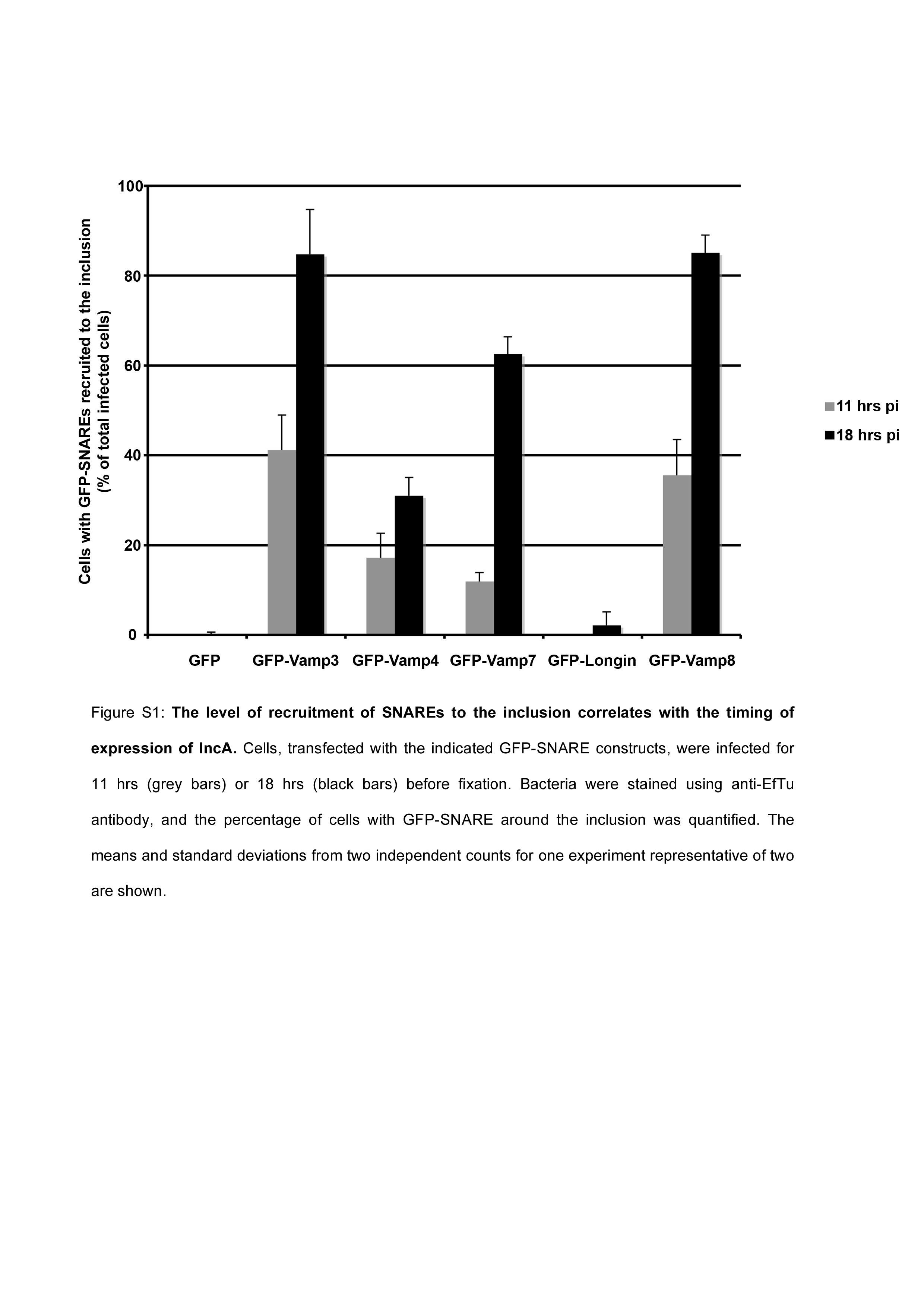

Supplement: Figure S1 — The level of recruitment of SNAREs to the inclusion correlates with the timing of expression of IncA. (2.00 MB TIF) [file ppat.1000022.s001.tif]

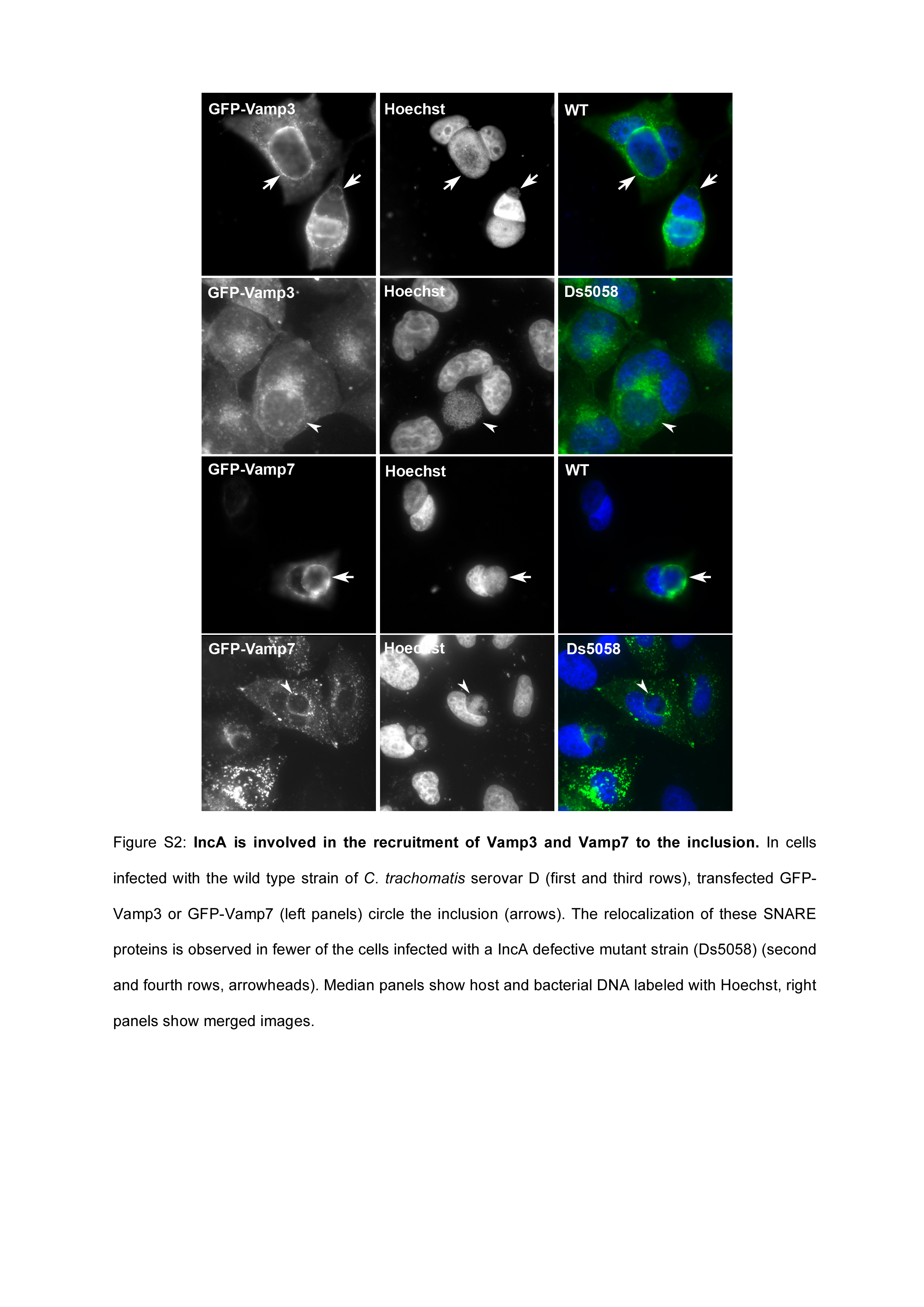

Supplement: Figure S2 — IncA is involved in the recruitment of Vamp3 and Vamp7 to the inclusion. (8.90 MB TIF) [file ppat.1000022.s002.tif]

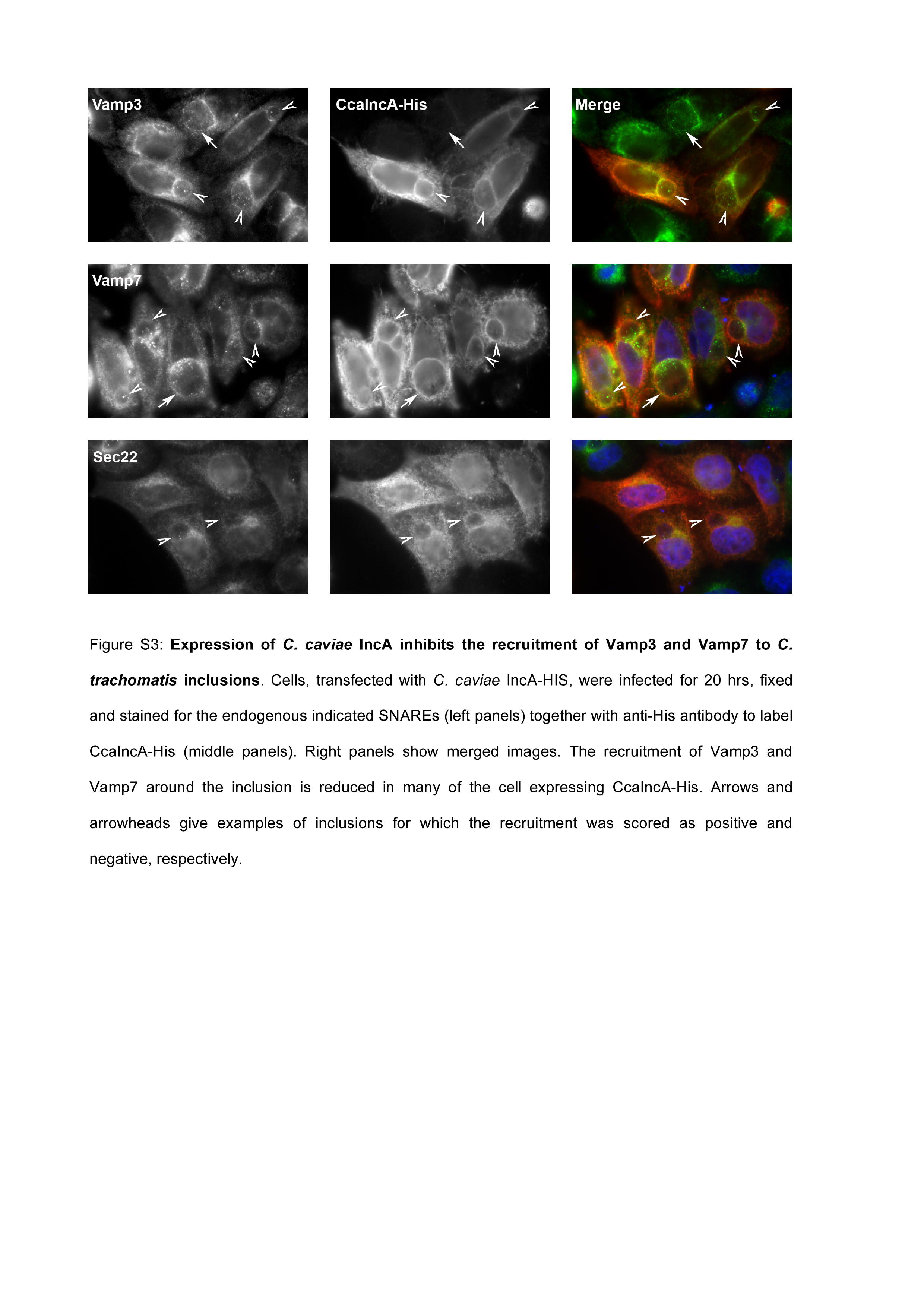

Supplement: Figure S3 — Expression of C. caviae IncA inhibits the recruitment of Vamp3 and Vamp7 to C. trachomatis inclusions. (9.99 MB TIF) [file ppat.1000022.s003.tif]

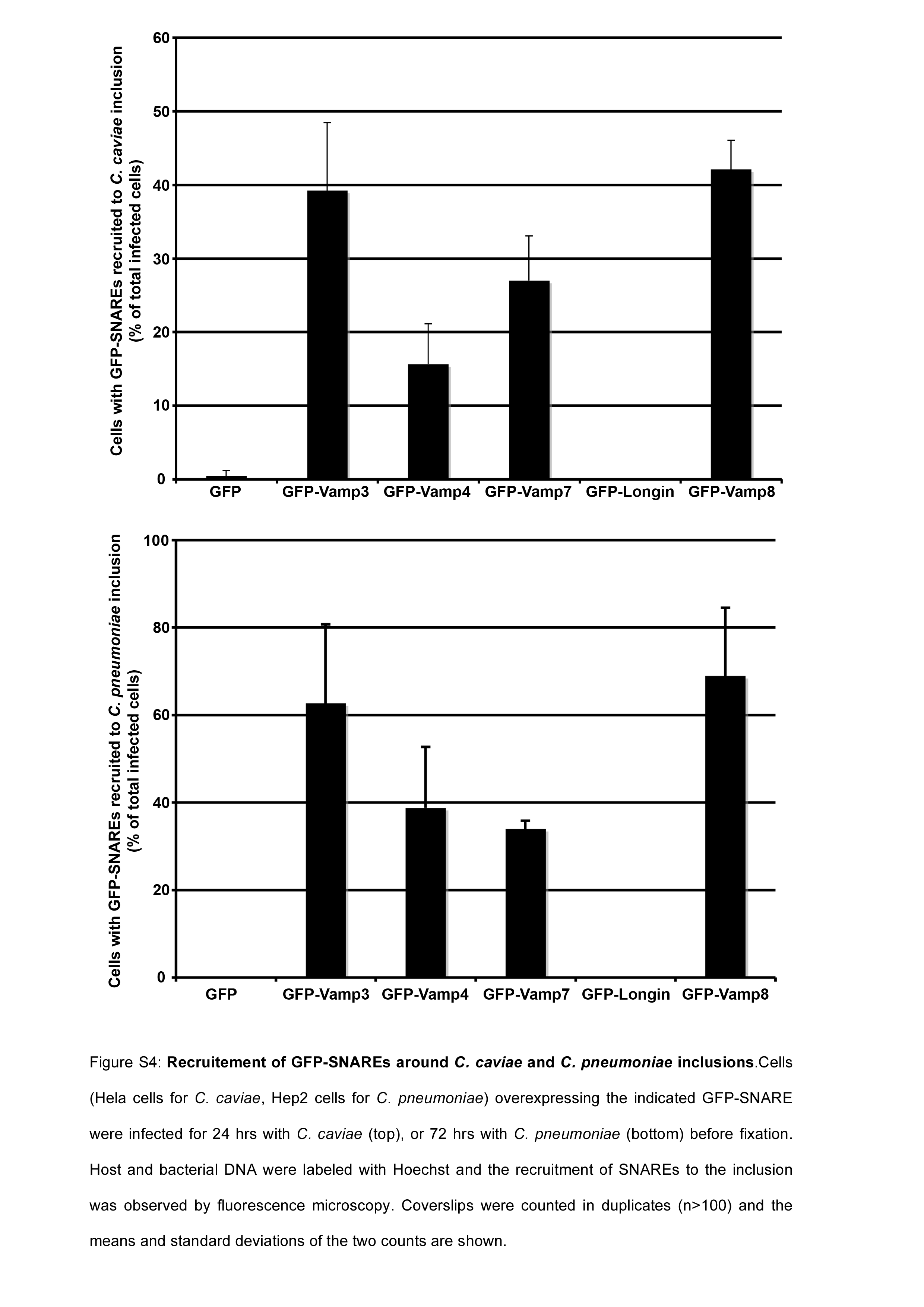

Supplement: Figure S4 — Recruitment of GFP-SNAREs around C. caviae and C. pneumoniae inclusions. (1.91 MB TIF) [file ppat.1000022.s004.tif]

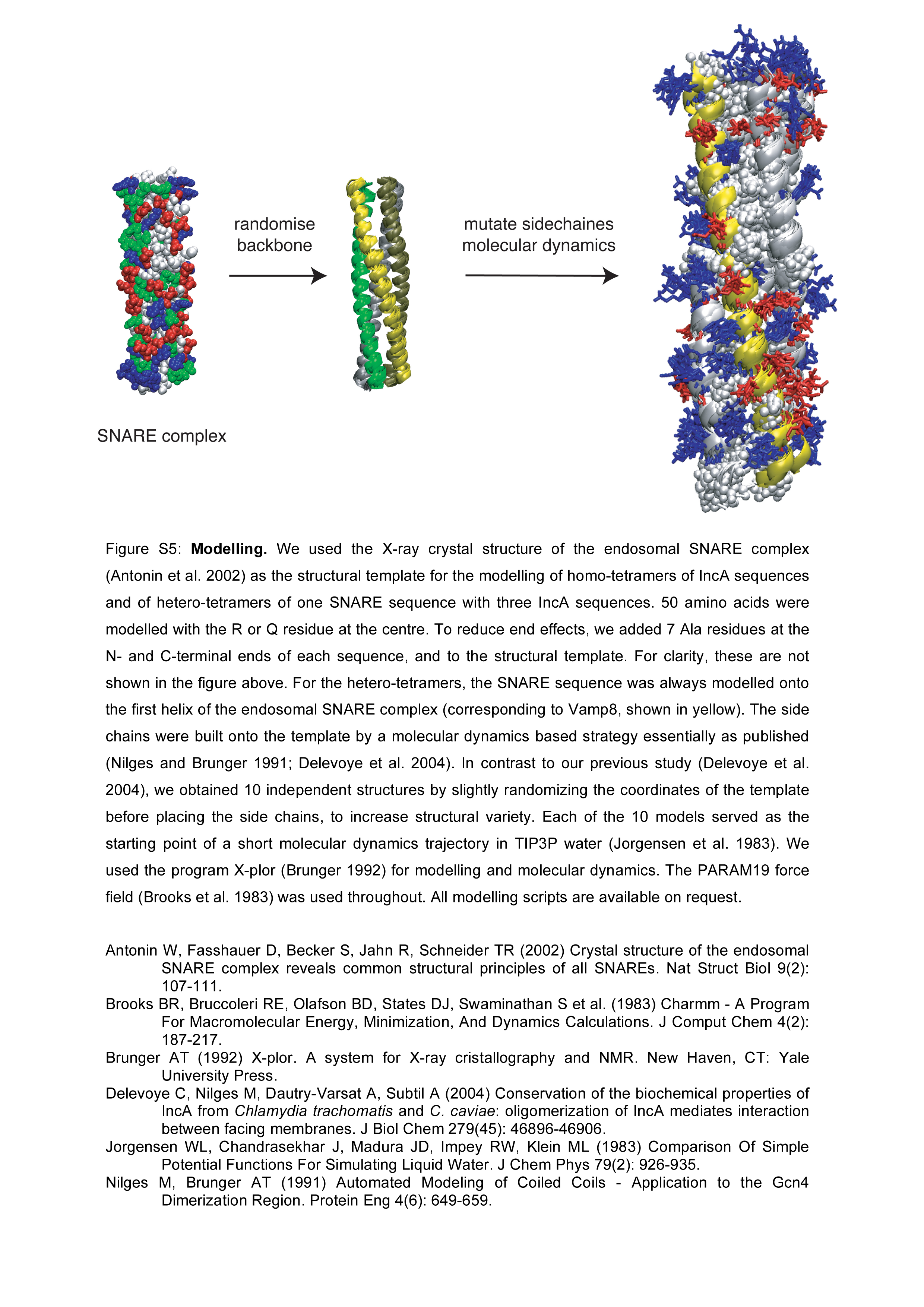

Supplement: Figure S5 — Modelling. (6.23 MB TIF) [file ppat.1000022.s005.tif]

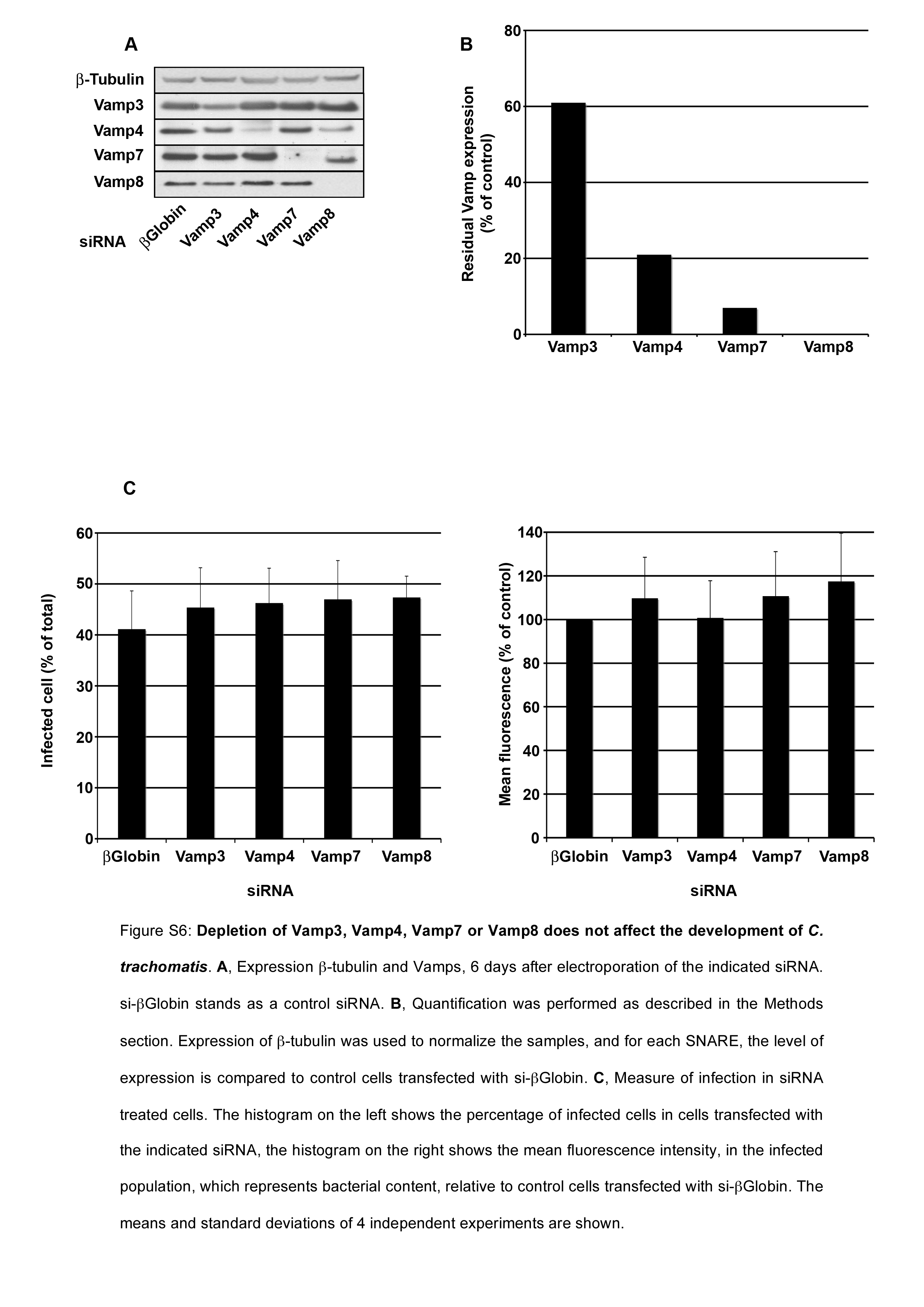

Supplement: Figure S6 — Depletion of Vamp3, Vamp4, Vamp7 or Vamp8 does not affect the development of C. trachomatis. (2.63 MB TIF) [file ppat.1000022.s006.tif]
